# Case Report: Surgical resection of high-grade extradural thoracic vertebral chondrosarcoma in a dog

**DOI:** 10.3389/fvets.2026.1767307

**Published:** 2026-04-13

**Authors:** Lauren Ho, Wei Zhi Lim, Jamie-Leigh Thompson

**Affiliations:** Queensland Veterinary Specialists, Brisbane, QLD, Australia

**Keywords:** chondrosarcoma, neurology, neurosurgery, oncology, surgery

## Abstract

**Introduction:**

This report describes the clinical presentation, diagnosis, treatment, and outcome of a dog with an extradural, non-compressive, high-grade thoracic vertebral chondrosarcoma (CSA).

**Case presentation:**

A 10-year-old, female spayed, 33-kg mixed-breed dog presented with a 96-h history of spontaneous vocalization and reluctance to use stairs. Neurological examination revealed normal ambulation without ataxia, along with marked resistance to manual ventroflexion and left lateral flexion of the neck. Magnetic resonance imaging (MRI) revealed a T1-weighted isointense, T2-weighted and STIR hyperintense, vividly contrast-enhancing mass with well-defined margins, located parasagittal to the right dorsal compartment of the T5 and T6 vertebrae, without evidence of neural invasion. Imaging and biopsy findings were consistent with vertebral CSA. Wide *en bloc* surgical resection was performed via a dorsal laminectomy with osteotomy of the spinous process using a bilateral paramedian approach to the T5–T7 vertebral column. Histopathological analysis confirmed a high-grade (grade III) chondrosarcoma with no histologic evidence of neoplasia at the bone margins. At the 2-week and 5-month recheck, the dog exhibited complete clinical resolution of CSA. Repeat computed tomography (CT) imaging performed 5 months postoperatively revealed no evidence of tumor recurrence.

**Conclusion:**

This case demonstrates that high-grade vertebral CSA can be successfully treated with wide surgical excision, particularly in the absence of neural invasion. However, long-term tumor surveillance is required.

## 1 Introduction

Chondrosarcoma (CSA) is a biologically malignant neoplasm characterized histologically by excessive production of a cartilaginous chondroitin sulfate matrix and the absence of osteoid production (1–3). CSA has been reported to occur primarily in the bones and cartilage of the appendicular and axial skeleton (e.g., nasal cavity, ribs, and long bones) and, to a lesser extent, extraskeletally (4–9). CSA is the second most common bony tumor of the vertebrae in dogs, and it is believed that it can originate *de novo* as a primary neoplasm or secondarily as a malignant transformation of a pre-existing benign cartilaginous growth (4, 9–11). Within the vertebrae, CSA classically involves the dorsal vertebral compartment (spinous process and apophyses) of several consecutive vertebrae (6). As the disease progresses, spinal canal invasion and neural compression occur, leading to myelopathy (4). The clinical signs of vertebral CSA are typically insidious, such as weeks to months of progressive neurological deficits, along with pain on spinal palpation of the affected region (6). High-field magnetic resonance imaging (MRI) is the diagnostic modality of choice, with characteristic findings of an extradural vertebral osteolytic and proliferative mass that is T2-weighted (T2W) hyperintense and T1-weighted (T1W) hypointense (6, 12).

A grading scale for axial and appendicular skeletal CSA in people has been adapted for dogs (13). According to the 2020 World Health Organization classification system, CSA is histologically classified as grade I to grade III based on the following histologic features: matrix production, architecture, degree of pleomorphism, cellularity, necrosis, and mitosis (13–15). In this grading scheme, each of the aforementioned features is assigned a numerical score (0–3), reflecting increasing severity, and mitotic activity is assessed by the number of mitotic figures per 10 high-power fields (HPF). Summed scores generate a total histologic score, which is then used to assign a tumor grade as follows: Grade I with a total histologic score of < 6 and mitosis = 0–1 or total histologic score = 7 and mitosis = 0; grade II with a total histologic score ranging from 7 to 10 and mitosis = 1 or 2; and grade III with a total histologic score ranging from 11 to 16 or mitosis = 3 (14).

Vertebral CSA presents unique therapeutic challenges due to its poor response to conventional chemotherapy and radiation therapy and the proximity to the spinal cord, making surgical intervention technically challenging (11, 16). In humans, wide *en bloc* surgical resection is the gold standard, and post-resection adjunctive therapy is not routinely recommended due to its unknown effect on survival time (9, 13). In veterinary patients, there have been reports of intralesional piecemeal resection as a treatment for vertebral CSA, but no cases have yielded complete tumor-free margins (6).

Prognosis in CSA in generally considered to be related to the completeness of surgical margins and histological grade, but large-scale, multi-institute studies are lacking. Farese et al. (14) have reported that dogs with grade I appendicular CSA had a median survival time (MST) of 6 years, and compared with those with grade III appendicular CSA, they had an MST of 0.9 years. However, a study reviewing rib CSA in dogs found no prognostic significance of the grading scheme applied, highlighting a difference between tumor location and prognosis (17).

Vertebral CSA is believed to follow a slow clinical course, but due to the rarity of this tumor location, CSA of vertebral origin is poorly characterized in the veterinary literature. The largest published dataset includes only six dogs, and all tumors were well-differentiated, low-grade lesions with low mitotic rates; however, all dogs had neural compression. Three of the six dogs underwent surgical intervention, and two of the three experienced regrowth and were euthanized within 5 months of treatment, suggesting an overall poor prognosis even in low-grade vertebral CSA cases.

In this case report, the authors describe the first case of extradural, non-compressive, high-grade thoracic vertebral chondrosarcoma in a dog, which was successfully managed with surgical resection.

## 2 Case presentation

A 10-year-old, female spayed, 33-kg mixed-breed dog presented to the surgical service of a private veterinary hospital in Australia with a 96-h history of spontaneous vocalization and reluctance to go upstairs. The dog had presented to the emergency department over the weekend, where it was treated initially as an outpatient with a single 0.2 mg/kg methadone dose given subcutaneously (SC). Pregabalin 75 mg was prescribed orally every 8 h, and a 100 μg/h transdermal fentanyl patch was placed. The clinical and neurological examination findings from that visit were unclear, but the patient was presumed to have cervical pain. Due to ongoing intermittent vocalization, the dog re-presented 12 h later and was admitted to the hospital for escalated analgesia overnight, prior to internal referral to the surgery service the following day for further investigation. The dog received 3 μg/kg medetomidine intravenously (IV) once and 0.2 mg/kg methadone IV every 4 h while hospitalized. Full bloodwork was performed. Complete blood count and serum biochemistry were within normal limits. C-reactive protein was 3.5 mg/L (reference range < 10 mg/L).

The emergency department reported that the dog was ambulatory on all four limbs without ataxia but exhibited marked resistance to manual ventroflexion and left lateral flexion of its neck. This finding was confirmed on examination by the surgical department. The cervical range of motion was well tolerated on dorsoflexion and right lateral flexion. The dog was able to move its neck freely, when encouraged, without vocalization. No repeatable pain response was elicited on direct, firm, cervical, or thoracolumbar spinal palpation. Mildly delayed proprioception was observed in the left pelvic limb, while all other limbs had no proprioceptive deficits. The dog’s mentation and behavior were normal, and cranial nerve assessment and spinal reflexes were normal. Given the acute onset of signs, resistance to manual cervical manipulation, and intermittent signs of pain reported by the owner, advanced imaging was recommended.

**Figure 1:**
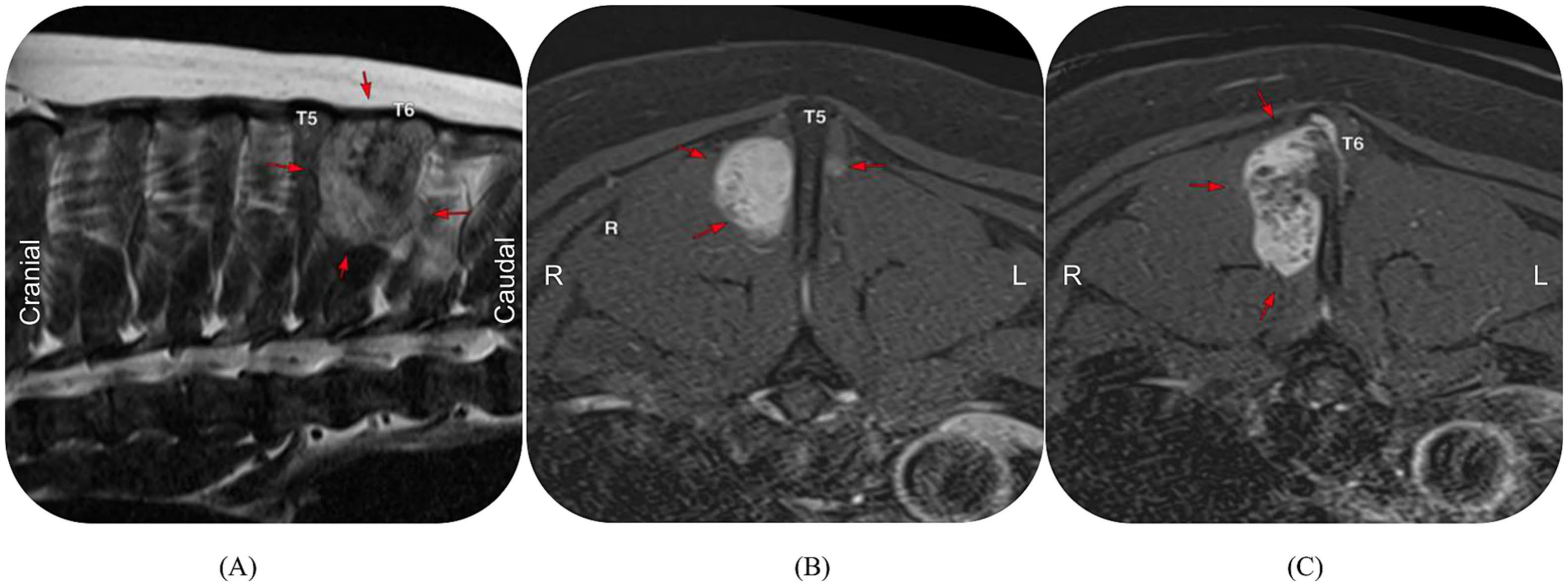
Magnetic resonance imaging (MRI) of the mid-thoracic spine. Sagittal T2-weighted view of T2–T7 vertebrae **(A)**. Transverse T2-weighted views of T5 **(B)** and T6 **(C)** vertebrae. These images demonstrate a hyperintense, vividly contrast-enhancing, well-margined mass (red arrows) parasagittal to the right dorsal compartment of the T5 and T6 vertebrae. There is no evidence of vertebral canal invasion.

**Figure 2:**
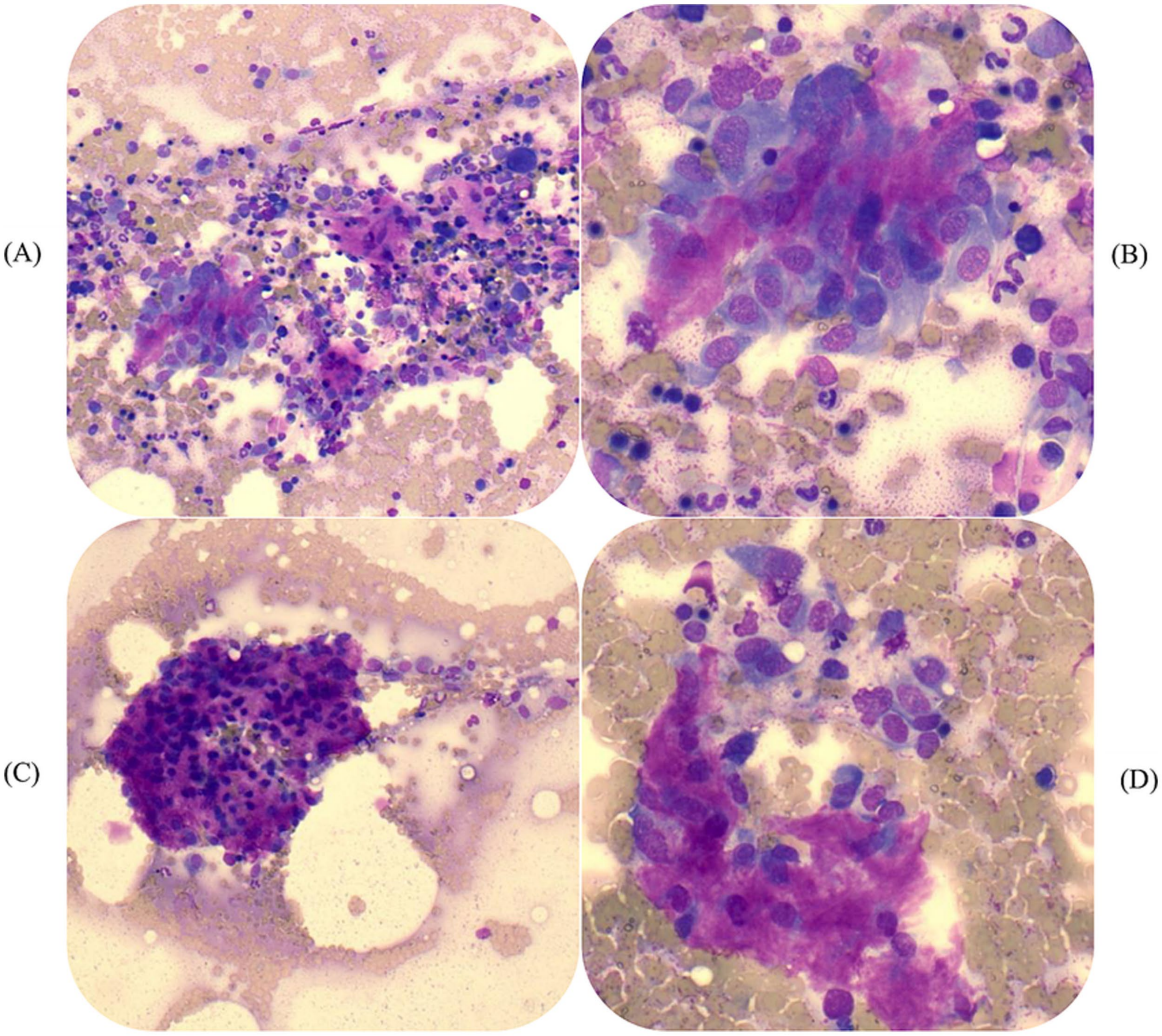
Photomicrographs of fine needle aspirate (FNA) cytology showing moderate to large numbers of individual and clumped angular to polygonal to elongated mesenchymal cells with mostly small ovoid nuclei, lightly stippled chromatin, and moderate amounts of light blue cytoplasm at 200x **(A)** and 500x **(B)** magnification. Hematopoietic cells are present. Neoplastic mesenchymal cell population in bright pink granular background matrix at 200x **(C)** and 500x **(D)** magnification (Wright’s stain).

MRI of the cervical and thoracic segments of the vertebral column was performed. MRI revealed a T1W isointense, T2W and STIR hyperintense, strongly contrast-enhancing mass with well-defined margins, located parasagittal to the right dorsal compartment (lamina, spinous process, and zygapophyses) of the T5 and T6 vertebrae (Figure 1). The mass measured 33 × 30 × 26 mm and extended approximately 1.5 cm dorsal to the T5 and T6 dorsal neural arch margins, with no evidence of invasion into the vertebral canal. The C6–7 annulus fibrosus was thickened dorsally without spinal cord compression. Chronic intervertebral disk protrusions were identified at the T13–L1 and L2–L3 disk spaces, causing mild left-sided spinal cord compression at the latter location. A fine-needle aspirate (FNA) of the mass was performed, which was consistent with sarcoma and showed evidence of bone remodeling. Cytologic evaluation revealed moderate to large numbers of individual to clumped angular to polygonal to elongated mesenchymal cells with mostly small ovoid nuclei, lightly stippled chromatin, and moderate amounts of light blue cytoplasm (Figures 2A,B). These cells were occasionally embedded within a bright pink granular background matrix (Figures 2C,D), and rare multinucleate cells with an osteoclastic appearance were noted. The dog was discharged with 15 mg/kg paracetamol orally every 8 h and 0.1 mg/kg meloxicam orally every 24 h, in combination with the pregabalin previously prescribed.

Two days later, a small incisional biopsy was performed. The patient was clipped, aseptically prepped, and positioned in sternal recumbency. A needle marker was placed in the dorsal spinous process of T6 under radiographic guidance. A mini right-sided dorsolateral approach to T6 was made, and the bony mass with a grossly dense fibrous capsule was identified. A single incisional biopsy was taken of the dorsal mass with a #15 blade, and hemostats were used to retrieve samples piecemeal from the same biopsy tract. The overlying soft tissue immediately dorsal to the mass was also sampled to assess for invasion. The incision was routinely closed in three layers. The biopsies were submitted for histopathological analysis. The dog was discharged on the same day with the same doses of paracetamol and pregabalin. Histopathological findings were consistent with chondrosarcoma, with no evidence of invasion into the adjacent soft tissue.

**Figure 3:**
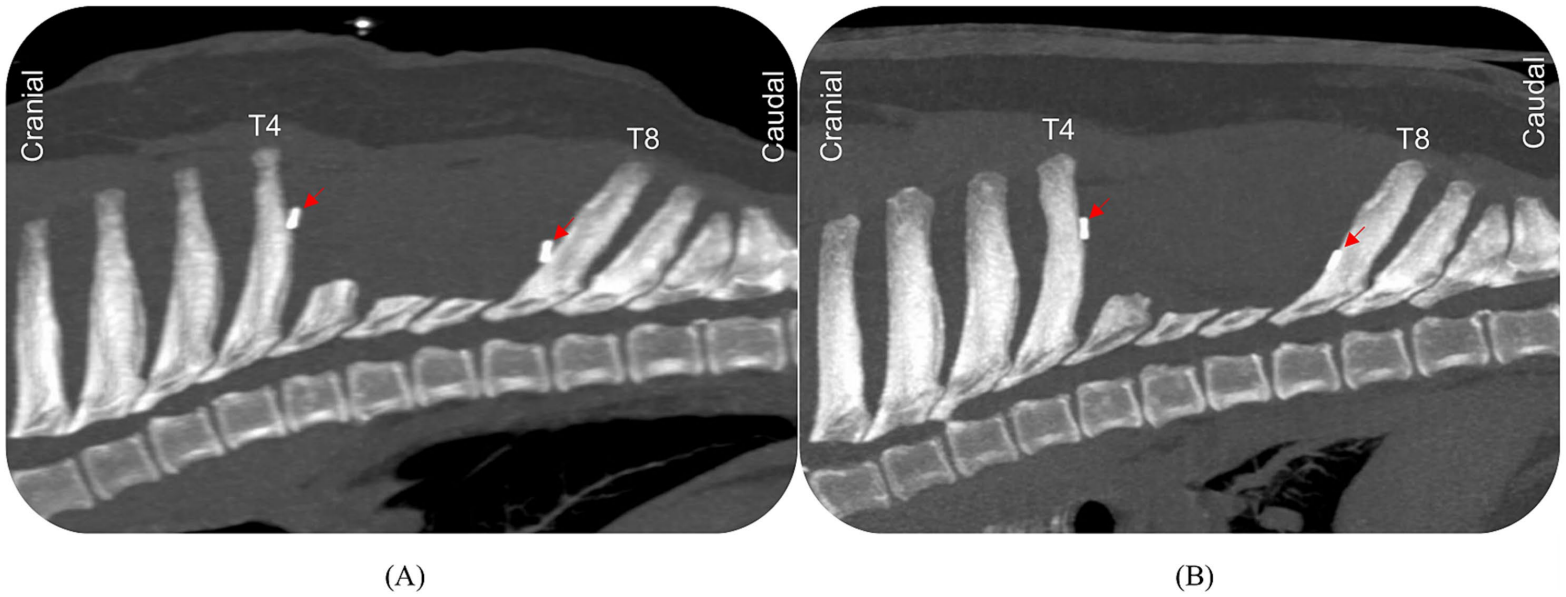
Computed tomography (CT) image of the mid-thoracic spine. Sagittal views of the T1–T11 vertebrae at immediate postoperative **(A)** and 5-month postoperative **(B)** time points. These images show radiographically complete mass excision via partial transection of the T5–T7 dorsal spinous processes. Hemoclips (red arrow) are placed at the cranial, caudal, and lateral margins in case of future radiation therapy. No radiographic evidence of tumor recurrence 5 months postoperatively.

Thirteen days after the biopsy, surgical excision of the mass was performed. The dog first underwent a computed tomography (CT) scan of the spine to confirm that there was no significant change in the lesion following the biopsy. Radiograph-guided placement of spinal markers at T6 and T7 was performed using a 25-gauge needle prior to transfer to the operating theater. The dog was positioned in sternal recumbency using sandbags and adhesive tape to secure it to the operating table. An elliptical cutaneous and subcutaneous incision was made with a 1-cm radial margin over the previous right-sided biopsy tract with a #10 blade. Once at the depth of the dorsal fascia, a #15 blade was used to perform a scalloped paramedian incision along the left side of the dorsal spinous processes of T5–T7. Elevation of the multifidus muscle off the left side of the spinous processes and articular facets was performed with osteotomes (6 and 8 mm) as per a routine hemilaminectomy approach. Elevation was continued until the rib heads were identified on the left side, and Gelpi retractors facilitated exposure. A similar approach was repeated on the right side to expose the rib heads of T5–T7, but a 1-cm radial cutaneous, subcutaneous, and fascial margin was maintained around the previous biopsy tract, and a 3- to 5-mm muscle margin was taken laterally around the entire tumor pseudocapsule. The capsule was not breached. This approach allowed isolation of the dorsal spinous processes of T5–T7 bilaterally. The large bony mass was grossly occupying the dorsal half of the spinous process of T6, extending cranially to the caudal border of T5. The lesion appeared firm and well-demarcated, with a dense pseudocapsule and no involvement of T7. There was no evidence of gross invasion into the spinal canal or intervertebral spaces. An oscillating saw was used to initiate a cut on the dorsal spinous processes of T5–T7, approximately 1–1.5 cm ventral to the ventral-most extent of the mass. The osteotomy was completed using an osteotome and a mallet. Due to the depth and angulation, the cut on T7 extended slightly deeper and resulted in the removal of a small (2 mm) portion of the dorsal roof/lamina, exposing the spinal cord. There was no contact with the spinal cord. The dorsal spinous processes of T5–T7 and the associated mass lesion and previous biopsy tract were excised *en bloc* and inked for histological assessment. The site was copiously lavaged with sterile saline, and surgical gloves and instruments were changed. Hemoclips were placed at the cranial, caudal, and lateral margins in case of future radiation therapy. Closure was performed in layers: the fascia was closed with 2–0 polydioxanone in a simple continuous pattern, the subcutaneous layer with 2–0 poliglecaprone-25 in a simple continuous pattern, and the skin with 3–0 poliglecaprone-25 in an intradermal pattern. A sterile dressing was placed over the surgical site while the patient was hospitalized. An immediate postoperative CT confirmed complete radiographic excision of the mass via partial transection of the T5–T7 dorsal spinous processes (Figure 3A).

Immediate postoperative analgesia was maintained initially with 0.2 mg/kg methadone IV every 4 h for the first 24 h and then administered as required for a further 24 h, in combination with 300 mg gabapentin orally every 8 h and 500 mg paracetamol orally every 8 h. The dog was discharged 2 days postoperatively with oral medications for 14 days. The dog remained ambulatory with no neurological deterioration throughout hospitalization and discharge, and the dog appeared comfortable at the time of discharge.

**Figure 4:**
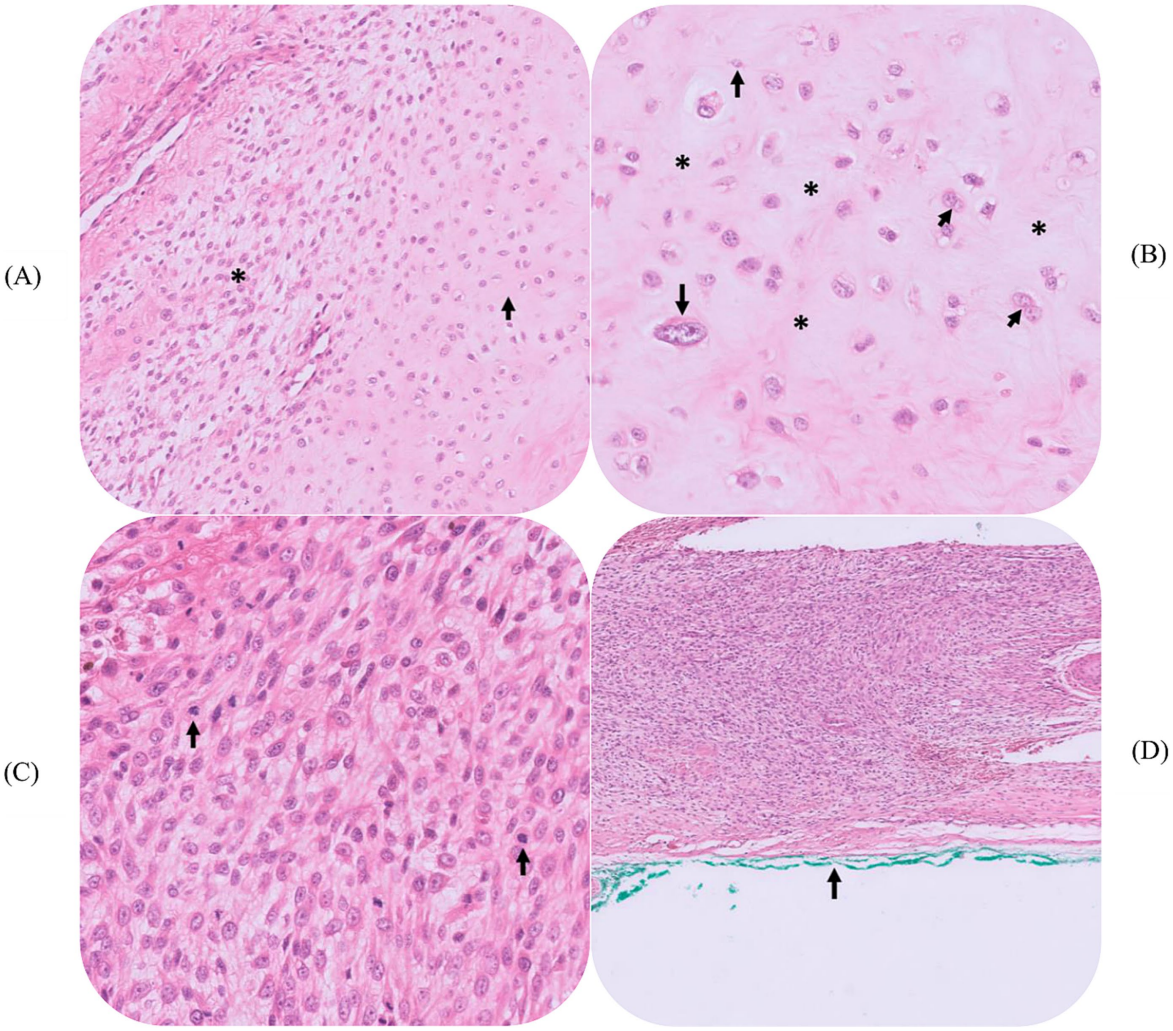
Representative histopathologic findings (hematoxylin and eosin). **(A)** Histopathology revealed the neoplastic mass to be composed of densely cellular streams of neoplastic spindle cells (asterisk) that merged with areas of chondroid differentiation and reduced cellularity (arrow) (200× magnification). **(B)** In the regions of chondroid differentiation, the neoplastic cells were embedded within a pale blue-gray to eosinophilic extracellular (chondroid) matrix (asterisks). There was scattered binucleation (short arrows) and areas of increased pleomorphism (long arrows) (400 × magnification). **(C)** The neoplastic cells exhibited mitotic activity, with up to 7 in 10 consecutive HPFs (400 × magnification). **(D)** Focally, in a section from the right lateral muscle margin, neoplastic cells abutted the inked histological margin (100 × magnification).

Histopathology results confirmed a diagnosis of high-grade (grade III) chondrosarcoma. This mass measured 27 × 20 mm on cut section and was composed predominantly of a moderately to densely cellular proliferation of spindle cells (Figures 4A,C). Multifocally, the neoplasm exhibited chondroid differentiation, with the neoplastic cells embedded within a pale blue-gray to eosinophilic chondroid matrix (Figures 4A,B). The occasional foci of endochondral ossification were also noted. The neoplastic cells had a moderate amount of eosinophilic cytoplasm, with plump, oval nuclei and one to two, often prominent nucleoli. There was mild to moderate anisocytosis and anisokaryosis, with occasional binucleation and up to 7 mitoses per 10 consecutive HPFs (Figures 4B,C). Occasionally, very small foci of tumoral necrosis were detected. Neoplastic cells focally abutted and multifocally extended to within 1 mm of the right lateral muscle margin (Figure 4D). Neoplastic cells did not extend to the remaining histological margins and, importantly, were not present in the sections examined from the bone margins.

At the 2-week recheck, the dog had returned to normal activity, and the owner reported no concerns, vocalization episodes, or reluctance to jump or walk upstairs. The neurological exam was normal, with no pain on direct spinal palpation or cervical manipulation, and the skin incision had fully healed. At the 3-month telephone follow-up, the dog remained clinically normal and active with no recurrent signs. Supplementary Figure S1 shows the clinical timeline for this patient. Repeat imaging to assess for tumor recurrence was planned for 6 months postoperatively, but it was limited to a CT scan due to financial constraints.

At the 5-month postoperative mark, the dog presented emergently with similar signs—reluctance to ambulate, intermittent spontaneous vocalization, and a new sign, low head carriage. Physical examination again revealed marked resistance to manual ventroflexion and left lateral flexion of the neck, and no pain on the remainder of spinal palpation. No lameness or ataxia was noted. Spinal CT revealed mild thickening of the dorsal annulus at C6–7 without spinal cord compression, consistent with previous MRI findings. There was no evidence of tumor recurrence or metastatic disease on the CT scan (Figure 3B). The dog was discharged with oral meloxicam and pregabalin at the same doses prescribed prior to surgery, for 14 days. All clinical signs had completely resolved, and physical examination was unremarkable at the 14-day re-examination. Active monitoring for recurrent signs and ongoing 6-monthly surveillance with spinal radiographs or cross-sectional imaging was recommended.

## 3 Discussion

To the authors’ knowledge, this is the first reported case of a high-grade thoracic vertebral CSA in a dog that was successfully managed with surgical resection. Previously published vertebral CSA cases in dogs have described well-differentiated, low-grade tumors associated with progressive neural compression and an overall poor outcome in the cohort, with the majority being euthanized by 5 months (5, 6). Our case expands the spectrum of reported tumor behavior for vertebral CSA. In an extra-skeletal caudal abdominal muscular CSA, survival time >196 days without evidence of metastases was achieved following the wide excision of a high-grade mesenchymal CSA in an 11-year-old dog (18). These cases demonstrate that, despite a high histological grade, a favorable functional outcome is achievable when effective local control is obtained.

In both humans and dogs, the presenting symptoms of vertebral CSA include pain, a palpable spinal mass, and neurological deficits when the mass extends into adjacent neural structures (6, 10, 11, 16). This case demonstrated resistance to ventroflexion and left lateral flexion of the neck while permitting range of motion in all other directions, and notably, no pain response was elicited upon direct spinal palpation. Additionally, the neurological deficits did not correspond directly with the tumor’s mid-thoracic location. The authors initially speculated that the observed cervical resistance may have reflected referred pain from the thoracic lesion, as there were no cervical compressive lesions identified on the MRI; however, it was possible that the CSA may have been entirely incidental, whereas the proprioceptive deficit identified in the left pelvic limb was likely explained by the concurrent compressive intervertebral disk protrusions at the T13–L1 or L2–3 disk spaces. Given the recurrence of clinical signs 5 months postoperatively, without evidence of tumor recurrence, it is improbable that the original clinical signs were attributable to the vertebral CSA. Given that the patient responded well on both occasions to conservative management, the authors speculate that the underlying cause may have been soft tissue in origin (i.e., muscle sprain/strain) or associated with the thickened C6–C7 annulus fibrosus reported on the MRI and CT scan.

Vertebral CSA has mostly been reported in large-breed dogs > 18 kg (4, 5, 8, 9). Therefore, the presence of unlocalized pain in a medium-to-large-breed dog should prompt further investigation into neurological pathologies, although concurrent neuropathies may confound neurolocalization. Early diagnosis at the point when minimal to no neurological deficits are present has been suggested to improve survival in dogs with vertebral tumors (10, 16). In a study of 20 dogs with vertebral osteosarcoma and fibrosarcoma, animals that presented with no neurological deficits had an MST of 330 days compared with an MST of 135 days for dogs with proprioceptive deficits or paralysis at presentation (16). The decision to pursue surgical treatment before neurological compromise was paramount for the successful clinical outcome in this case.

Radiographs were not performed in this case but may be beneficial as a lower-cost screening tool for bony neoplasia of the vertebrae in large-breed dogs with non-specific symptoms. Expected radiographic signs include calcification, deep endosteal scalloping, cortical disruption, periosteal reaction, a soft tissue mass, and a moth-eaten pattern of lysis (5, 6, 9–11). Cross-sectional imaging proved useful for assessing extraosseous extension and for surgical planning. The MRI features of an extradural vertebral osteolytic and proliferative mass that is T2W hyperintense and T1W hypointense were consistent with previously described characteristics of vertebral CSA (6, 12). Roynard et al. (6) suggested that specific MRI findings may help predict tumor grade prior to histological confirmation. The lobulated, T2W hyperintense regions within these tumors correspond to the high water content of the neoplastic cartilage matrix, whereas foci of T1W hypointensity represent areas of endochondral ossification within the mineralized extracellular matrix (11). This heterogeneous appearance mirrors the histologic architecture of CSA, where different regions of the same tumor show variable maturation and differentiation (11). In low-grade CSA, distinct heterogeneity reflects well-differentiated CSA. In the present case, the lack of a clear distinction between the aqueous cartilage matrix and calcification foci on MRI suggests a poorly differentiated neoplasm, consistent with the histologic diagnosis of high-grade (grade III) CSA. Therefore, a more homogeneous T2W hyperintense vertebral mass may indicate high-grade CSA, further supporting the use of MRI findings for presumptive tumor grading. The preoperative CT findings in this case aligned with the previous MRI findings, suggesting that a CT scan may be a reasonable alternative in cases where MRI is not affordable or readily available.

Extrapolating from human data, vertebral CSA is considered resistant to conventional chemotherapy and radiation, making surgical resection the treatment of choice (11). Beyond the histological grade, prognosis depends on surgical resectability. Recurrence is common when the tumor invades the epidural space, as this limits the ability to achieve clean margins. *En bloc* resection with wide, disease-free margins offers the best local control, with recurrence rates in humans reported to be 3–8% (19–21). In contrast, intra-lesional piecemeal resection is associated with nearly 100% recurrence, typically 3–5 years postoperatively in humans and as early as 3 months in dogs (6, 11, 19–22). Roynard et al. reported the cases of six dogs with neural invasion at the time of diagnosis of vertebral CSA, four of which were euthanized for severe neurological deficits, attributable either to the primary tumor or tumor recurrence following piecemeal resection (6). In contrast, the present case achieved local control and rapid neurological recovery following wide *en bloc* resection without adjunctive therapy. The tumor location and the absence of spinal cord compression were believed to be key contributors to the favorable clinical outcome observed and may represent favorable prognostic factors in cases of high-grade vertebral CSA. Larger studies are needed to confirm prognostic indicators for vertebral CSA in veterinary patients. In humans, the Weinstein–Boriani–Biagini (WBB) staging system allows the topographic division of the vertebral body into 12 zones and five layers to accurately record tumor location for staging and to plan the feasibility of oncological resection (22). No specific guidelines to classify vertebral tumor location have been developed for dogs.

Given the lack of neural invasion in the present case, *en bloc* resection with curative intent was attempted. An FNA and an incisional biopsy were performed initially to characterize tumor biology and guide surgical planning, such as the determination of appropriate resection margins. During wide *en bloc* resection, a radial incision was made to encompass the previous biopsy tract from the skin to the fascia. As a core principle of surgical oncology, an inclusion of the biopsy tract is recommended to reduce recurrence risk by preventing residual tumor cell implantation (21). A dorsal laminectomy with osteotomy of the spinous process using a bilateral paramedian approach to the cranial thoracic vertebral column facilitated easy access to the neoplasm during biopsy and surgical resection. A similar unilateral approach has been described in 14 dogs for the decompression of subarachnoid or synovial cysts and intradural–extramedullary neoplasia, exposing up to 75% of the dorsal and lateral compartments of the spinal cord (23). In the present case, secondary spinal stabilization was not required, no surgical complications were encountered, and the dog had no evidence of neurologic deterioration postoperatively. Treatment options for primary vertebral CSA should be further evaluated in a larger group of dogs to determine if survival time can be prolonged with *en bloc* resection compared with other therapies.

Follow-up with the owner and physical examination at 5 months postoperatively documented complete resolution of vertebral CSA, and a repeat CT scan revealed no evidence of recurrence. The excellent short- to mid-term outcome in this study underscores that tumor grade and prognosis should be interpreted alongside completeness of resection, degree of neural compression, and anatomical location. Despite the ability to remove the tumor *en bloc*, tumor cells appeared to abut the right lateral muscle (soft tissue) margin histologically. Given that the CSA is primarily a tumor of the bone, it may be interpreted that a clean bone margin would be sufficient to report a “clean” cut; however, the consequence of tumor cells abutting the soft tissue margin is unknown in the context of vertebral CSA in dogs. The lack of long-term follow-up in this case is a major limitation of this report, and the likelihood of tumor recurrence in the future is uncertain. Continued imaging surveillance remains warranted given the higher-grade diagnosis and lack of published cases. Active monitoring at home and biannual rechecks with imaging have been recommended to screen for potential tumor recurrence.

The relative utility of CT compared with MRI for detecting vertebral CSA has not been systematically evaluated in dogs. Roynard et al. (6) described CT findings in one dog as an osteoproliferative and osteolytic lesion affecting the dorsal vertebral arches. Given that vertebral CSA originates from osseous structures, CT is likely sufficient to identify tumors in many cases; however, its ability to fully characterize tumor extension and its relationship to the spinal cord is limited compared with MRI. Since the study’s outcome may be influenced by the extent of spinal cord compression or invasion, this case emphasizes that detailed assessment of cord involvement serves as an important prognostic indicator.

In conclusion, the authors believe this to be the first case of a high-grade vertebral CSA in the veterinary literature and one that was successfully treated with wide surgical excision. A key factor for success was the tumor location and the absence of neural invasion, as identified on MRI. The use of a modified dorsal laminectomy was effective for this tumor location and resulted in no postoperative complications. Both short-term and medium-term outcomes were excellent, with immediate return to function and no clinical signs or CT evidence of tumor recurrence at the 5-month follow-up. Long-term tumor surveillance with repeat imaging is warranted.

## Data Availability

The original contributions presented in the study are included in the article/Supplementary material, further inquiries can be directed to the corresponding author/s.

## Supplementary material

SUPPLEMENTARY FIGURE S1 Figure showing a timeline of key clinical events.
