# Supplementary figures and images for: Case Report: Surgical resection of high-grade extradural thoracic vertebral chondrosarcoma in a dog
Source: Front Vet Sci. 2026 Apr 13;13:1767307. doi: 10.3389/fvets.2026.1767307 (PMC13111167; doi:10.3389/fvets.2026.1767307)

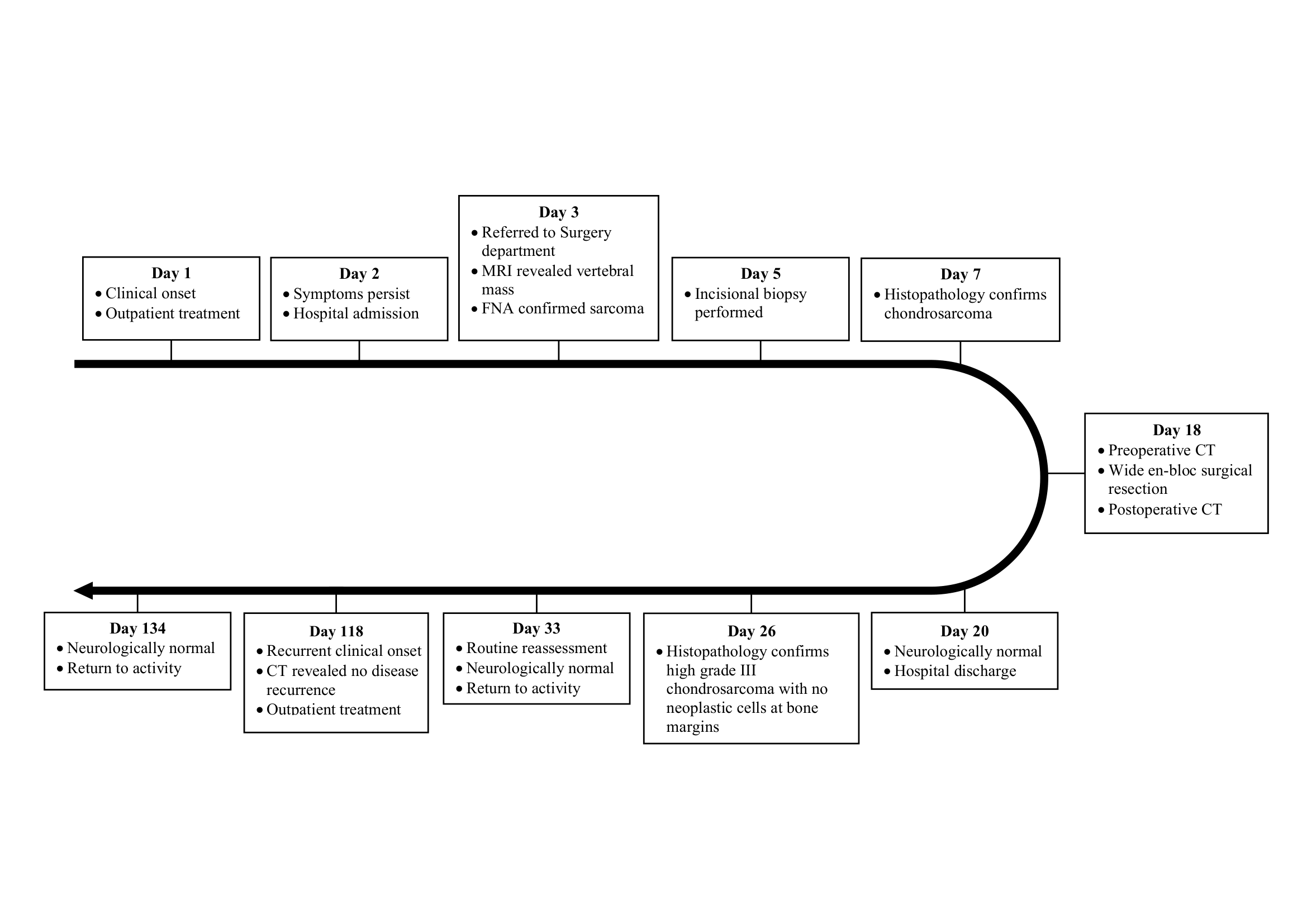

Supplement: SUPPLEMENTARY FIGURE S1 — Figure showing a timeline of key clinical events. [file Image_1.tiff]
